# Humidity Resistant Biodegradable Starch Foams Reinforced with Polyvinyl Butyral (PVB) and Chitosan

**DOI:** 10.3390/polym16233402

**Published:** 2024-12-03

**Authors:** Apoorva Kulkarni, Jakob Emrich, Ramani Narayan

**Affiliations:** Department of Chemical Engineering and Materials Science, Michigan State University, East Lansing, MI 48824, USA; kulkar75@msu.edu (A.K.);

**Keywords:** biodegradable foams, chitosan, starch, extrusion foaming, humidity resistant

## Abstract

In this study, water-insoluble, moisture-resistant starch foams were prepared using an optimized one-step extrusion-foaming process in a ZSK-30 twin screw extruder. The extrusion parameters, including temperature, screw configuration, die diameter, water content, and feeding rates, were optimized to achieve foams with the lowest density and controlled expansion. A screw configuration made up of three kneading sections was found to be the most effective for better mixing and foaming. Polyvinyl butyral (PVB) acted as a plasticizer, resulting in foams with a density of 21 kg/m^3^ and an expansion ratio of 38.7, while chitosan served as a nucleating agent, reducing cell size and promoting a uniform cell size distribution. The addition of PVB and chitosan reduced the moisture sensitivity of the foams, rendering them hydrophobic and water-insoluble. The contact angle increased from 0° for control foams to 101.5° for foams containing 10% chitosan and 10% PVB. Confocal laser scanning microscopy (CLSM) confirmed the migration of chitosan to the foam surface, enhancing hydrophobicity. Aqueous biodegradation tests, conducted at 30 °C in accordance with ISO 14852 standards, demonstrated that despite enhanced moisture resistance, the foams remained readily biodegradable, achieving approximately 80% biodegradation within 80 days. These modified starch foams present a sustainable solution for packaging and insulation applications that demand long-term humidity resistance.

## 1. Introduction

Biodegradable and biobased plastics have gained significant attention as potential replacements for their petroleum-based counterparts. Traditional foams such as polystyrene, polyethylene, polyvinyl chloride, and polyurethane can take centuries to fully degrade, causing prolonged negative impacts on soil, water, and other environments [1,2]. This underscores the critical need for developing degradable plastics that can serve as viable alternatives to traditional plastics. Currently, the foam packaging market is under increasing pressure from environmental regulations and disposal mandates that call for better carbon management and new end-of-life initiatives. There is an urgent need to reduce the carbon footprint of the package and provide environmentally responsible end-of-life disposal alternatives [3]. Biobased and biodegradable starch foams are recognized as promising alternatives to polystyrene foams for applications in packaging, insulation, and medical fields [4,5]. These foams can be produced through various methods, including extrusion, baking, microwave expansion, freeze-drying, etc. [6]. Among these, reactive extrusion offers distinct advantages, such as rapid reaction times, enhanced heat and mass transfer, improved mixing, and solvent-free processing [7].

The extrusion process has been widely utilized in the food industry for several years to produce various modified starch products, including expanded snack items. However, starch inherently is non-thermoplastic; it neither softens nor flows. Its thermal degradation temperature is lower than its melting point. Plasticizers such as water, glycerol, and sorbitol are commonly added to make the starch processable and flow like a thermoplastic [8]. These plasticizers disrupt the intermolecular hydrogen bonds between the starch molecules and destroy their crystalline structure.

Starch foam extrusion is a two-step process. In the first step, starch, additives, and water are introduced into the extruder. Under the influence of high temperature, shear, and pressure, water interacts with starch, disrupting its granular structure. Then in the second step, as the mixture exits the die, a sudden drop in pressure causes water to evaporate and form the cellular structure of the foam [9,10,11]. Foam formation occurs in three distinct phases: nucleation, bubble growth, and stabilization. The nature and cellular structure of the foam are influenced by various extrusion conditions, such as screw configuration, starch type, temperature, water content, and presence of other additives, such as blowing or nucleating agents [6].

Starch is an abundantly available polysaccharide that is nontoxic, easily biodegradable in almost any environment, inexpensive, and possesses many superior properties. However, starch foams have some shortcomings, such as very low mechanical strength [12], poor moisture resistance, and immediate solubility in water [4,13]. They are sensitive to moisture, and they tend to shrink and lose their cell structure in high-humidity conditions. Furthermore, condensation of water on the foam surface can dissolve them, compromising their mechanical integrity and limiting their use in packaging and insulation applications. Extensive research is being conducted to address these issues by incorporating various additives. This study focuses on designing and engineering moisture-resistant starch foams to overcome these challenges.

Several hydrophobic polymers such as polyhydroxy amino-ether (PHAE), PLA, PBAT, and cross-linking agents like glyoxal, glutaraldehyde, and citric acid have been investigated as additives to enhance the humidity resistance of starch foams [4,13,14,15]. However, production of PHAE was discontinued in 2002, and currently, it is not available commercially. Also, one of the building blocks of PHAE is bisphenol A (BPA), which is a suspected endocrine disrupter [16], and its use in “green” material negates the “green” nature of the product. Cross-linking agents like glyoxal are toxic and hazardous and hence cannot be used for making foams for medical and food contact purposes [17]. Using starch esters instead of native starch has been explored, but this approach was deemed economically unfeasible [18,19]. Another method, dip-coating starch foams with hydrophobic polyesters, has also been tried to improve moisture resistance, but this two-step approach requires extra time and equipment, limiting its scalability and practicality for industrial applications [20].

**Figure 1:**
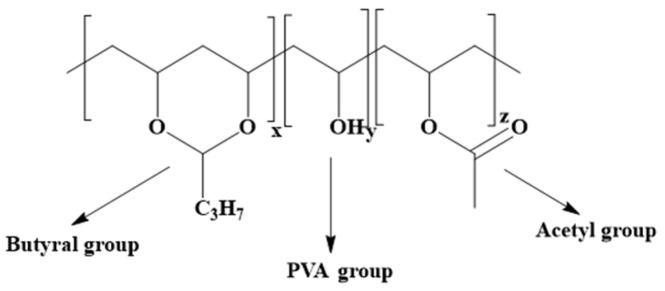
Structure of polyvinyl butyraldehyde (PVB).

This study investigates the use of readily available polyvinyl butyral (PVB) and chitosan as environmentally friendly additives to enhance the humidity resistance of starch foams. PVB is a thermoplastic polymer synthesized via a condensation reaction between polyvinyl alcohol and butyraldehyde. It is hydrophobic, nontoxic, and suitable for direct food contact applications [21]. PVB is predominantly used for applications that require strong binding, adhesion, flexibility, and moisture resistance. Figure 1 shows the structure of PVB. PVB is commonly incorporated into composites and films to improve moisture resistance and hydrophobicity [22,23]. However, to the best of our knowledge, there are no prior studies exploring the use of PVB to enhance the moisture resistance of starch foams.

Chitosan, derived from the second-most abundant polymer chitin, exhibits excellent biocompatibility and biodegradability. It is nontoxic and demonstrates noteworthy biological activities, coupled with excellent strength and elongation properties. Dang et al. reported that, when blended with starch, chitosan migrates to the surface of the films and decreases the hydrophilicity of the materials [24]. Hence, chitosan was used here along with PVB to study its effects on the moisture resistance properties of foams.

In this work, extrusion parameters, such as screw configuration, temperature profile, die diameter, rpm, and feed ratios, were optimized. The effects of incorporating PVB and chitosan as additives were evaluated to enhance hydrophobicity and render the foams water-insoluble. The physio-mechanical properties of the foams, including density, expansion ratio, and compressive strength, were evaluated and compared with commercial starch foams. Key properties, such as moisture sensitivity and contact angle, were also reported for the various formulations studied. Finally, the end of life of these foams in aqueous environment was investigated. These foams have potential applications in packaging or insulation where long-term moisture resistance and mechanical properties are required.

## 2. Experimental Section

### 2.1. Materials

High amylose corn starch was obtained from National Starch and Chemicals (Indianapolis, IN, USA) with equilibrium moisture content of 12% (*w*/*w*). Water served as both plasticizer and blowing agent. Polyvinyl alcohol (PVOH), used as an additive to make control foams, was supplied from Kuraray America (Houston, TX, USA), Inc. under the trade name Mowiol 40–88 (M_w_~205,000 g/mol). Polyvinyl butyral (PVB), also from Kuraray America Inc., was provided under the trade name Mowital B 60HH (non-volatile content ≥ 97.5, residual polyvinyl alcohol content 12–16%, residual polyvinyl acetate content 1–4%, dynamic viscosity 120–280 mPa.s, Tg 65 °C). Talc (magnesium silicate), used as nucleating agent, was obtained from Luzenac (Timmins, On, Canada). Chitosan, provided by Primex EHF (Siglufjordur, Iceland) under the trade name ChitoClear^®^ 42010-cg110 75cp, was derived from Fresh North Atlantic Shrimp Shells (Pandalus borealis). It had a degree of deacetylation >75%, a viscosity of 75cP, solubility > 99%, and dry matter > 90%.

### 2.2. Preparation of Starch-Based Foams via Foam Extrusion Process

**Figure 2:**
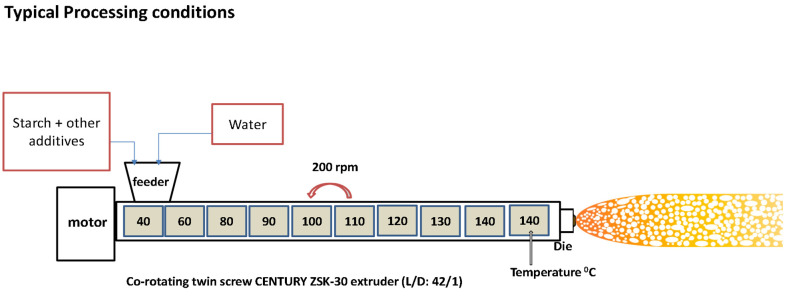
Experimental setup for foam extrusion.

The laboratory starch foam extrusion was conducted using a co-rotating twin screw extruder (Century ZSK-30, Century Extrusion, Traverse city, MI, USA) with an L/D of 42:1 (Figure 2). A peristaltic pump (model C.P. 78017-10, Ismatec) was used for injecting water into the extruder, and accurate single-screw feeders were used for feeding starch and additives. A screw speed of 180–200 rpm was maintained for foam noodle extrusion. Three different strand dies with 3.5, 4.3, and 8 mm diameters were used for making foams. Both the peristaltic pump and single-screw feeders were calibrated for various feed rates of water and starch to maintain process consistency.

During startup, the starch feed rate was kept lower (50–60%) than the final feed rate, while the water feed rate was maintained at a higher level than the final water feed rate (approximately 20–25% of starch feed rate). As the mixture began to extrude from the die, the starch feed rate was gradually increased, and the water feed rate was decreased until the product started foaming consistently. For most runs, a starch feed rate of 9 kg/h was found to be optimal, with a water flow rate of 6–8% of the starch feed rate for consistent foam production. The starch used had an equilibrium moisture content of 12%, resulting in a total water content of 18–20% on a dry basis [4,13,25]. Additives such as talc, PVOH, and chitosan were incorporated to control the foam cell size, distribution, and overall surface characteristics of the foams. Table 1 provides a comprehensive summary of all the runs, including the various additives used for foam extrusion and their respective concentrations.

### 2.3. Characterization and Analysis

All the samples were conditioned for at least 72 h at 50% relative humidity and 23 °C as per ASTM D4332 prior to any testing.

*Density*: The foam densities were determined by calculating the mass-to-volume ratio of the samples according to ASTM D3575. The dimensions were measured using a Vernier caliper with a precision of ±0.01 mm. For the cylindrical foam samples, measurements were collected at various time intervals during each run, with 10 measurements per sample to determine an average diameter and density.

*Expansion ratio*: The expansion ratio of the cylindrical foam was calculated by dividing the foam’s cross-sectional area by the cross-sectional area of the die (in mm^2^). The reported value represents the average calculated from ten samples for each formulation.

*Water solubility test:* A 0.5 g sample of starch foam was placed in water and stirred. Pictures were taken at specific intervals (1 min, 5 min, 30 min, 3 h, and after 2 days) to observe whether the foams dissolved in water or not.

*Surface wetting test*: Foam noodles were immersed in water, removed, and left undisturbed for five hours. After 5 h, photographs were taken to determine the degree of disintegration of the foam noodles.

*Scanning electron microscopy (SEM):* A JEOL 6610 LV scanning electron microscope (JEOL Ltd., Tokyo, Japan) was utilized to analyze the cell size distributions and surface morphology of the samples. The foam samples were sliced with a razor blade, mounted on aluminum stubs, and then coated using a sputter coater before examination. To avoid damaging the cell structures, imaging was conducted at a reduced voltage of 7 kV. Images were captured at magnifications of 15× and 20×. ImageJ software (Java 1.8.0_421) was used to calculate the average cell size and distribution within the foams.

*Moisture sorption analysis:* Three samples of each foam formulation were placed in a 95% relative humidity chamber at 25 °C. The sample’s weight and dimensions were monitored at regular intervals until they reached a steady state value.

*Absorption isotherms:* The moisture adsorption isotherms of starch foams were investigated under varying relative humidity (RH) conditions. Six distinct saturated salt solutions were prepared and placed at the bottom of desiccators to achieve the RH values from 11% to 95%. The starch foams were oven-dried at 90 °C for 48 h, weighed, and then placed in the closed desiccators for a definite amount of time. Changes in the moisture content of the starch foams as a function of time for different foam samples were recorded to generate the adsorption isotherms. Table 2 shows the salt solutions used to create the controlled humidity environments.

*Confocal microscopy:* Chitosan-containing foam samples were visualized using Fluoview FV1000 CLSM inverted-type microscope (Olympus, Tokyo, Japan). A fluorescein isothiocyanate (FITC)/water solution (50 mg/mL) was prepared, and the starch foam samples were soaked in it for about 1 min. Then the samples were taken out, washed with distilled water to remove any excess dye, and mounted on glass slides for imaging. A UPLFLN objective with 10× magnification and numerical aperture of 0.3 was used for capturing the images. The chitosan was labeled with FTIC for getting green fluorescence [24]. Excitation at 488 nm was provided using argon lasers. Emission filters SDM560 and BF505–525 were used for collecting green fluorescence in channel 1. Then a Z series was collected over the thickness of 388 um with a Z-step size of 6.93 microns. Maximum intensity projection image (MIP) was generated and saved using Olympus FLUOVIEW 4.2 software. Kalman average of four images was used to reduce the image’s background noise. The foam samples were analyzed for two positions in the sample: the surface and middle section of the foam. The fluorescent areas in the images were quantified using ImageJ software (Java 1.8.0_421).

*Contact angle measurements:* Contact angle measurements were performed using a DSA30S contact angle goniometer (Krüss GmbH, Hamburg, Germany) at 25 °C. Water droplets were dropped precisely on the surface of the foams using a micro syringe, and contact angles were measured using Advance software version 1.11 (Krüss GmBH, Germany). An average of four to five measurements were taken for a single sample at different positions. The measured values were reported 30 s after the deposition of water droplets. Changes in the contact angle after 1 min of deposition were also observed.

*Compressive strength and resiliency:* Compressive strength of the foam noodle specimens was measured according to ASTM D1621 using an Instron testing machine [26]. The cylindrical foam samples were placed between the compression plates, and an initial load of 0.5 N was applied. Then the samples were pressed at a rate of 19 mm/min for a deformation of 13% or yield point, whichever occurs first. The maximum compression load was recorded. Compressive strength was recorded by dividing the maximum compression load by the cross-section area. Then the plate was returned to its original position, and a second relaxation load was measured after 60 s. Percent resilience was calculated as the force required for the second compression divided by the first. All the readings were obtained as an average of five to seven samples for each formulation.

*Biodegradability:* The biodegradability of two foam samples was assessed in an aqueous environment under aerobic conditions at 30 °C. The samples included: (1) starch foam with no additives as control foam, (2) PVB and chitosan, and (3) cellulose as positive control. A respirometric mineralization test system, designed to measure CO_2_ evolution, was set up according to International Standard ISO 14852 [27]. The system included blank, positive reference (cellulose) and the test materials for all the runs. All the samples, blanks, and references were tested in duplicates. Detailed information on the setup and testing procedures can be found in our reference [7]. Cumulative CO_2_ evolution plots and % biodegradation vs. time were generated for all the samples and blanks to compare the biodegradability of the foam samples.

## 3. Results and Discussion

### 3.1. Foam Extrusion Process Optimization

Various parameters in the extrusion process, including screw configuration, temperature, water content, screw speed, and die diameter, were optimized to achieve a consistent, uniform foam production process.

**Figure 3:**
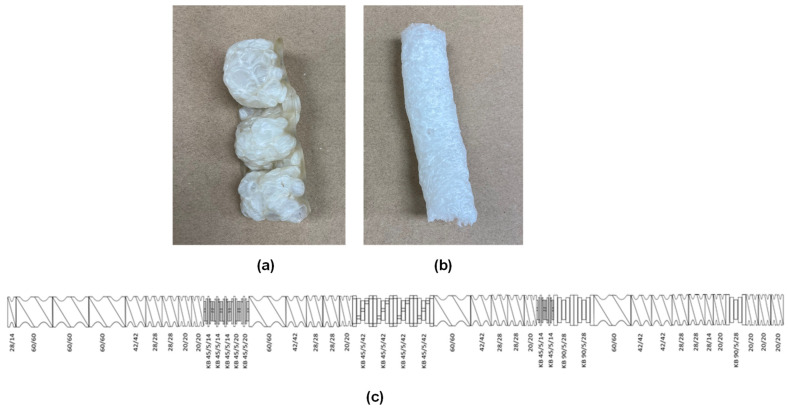
Foams obtained from (**a**) screw configuration #1, (**b**) screw configuration #2, and (**c**) second screw configuration used for foam production.

*Screw configuration:* The screw configuration plays a crucial role in the foaming process. During foaming, the crystalline structure of starch granules is disrupted, with water acting as a plasticizer. Efficient mixing of water and additives is essential to form a homogenous phase, ensuring consistent quality foams. Therefore, the screw configuration was specifically designed to meet these objectives. Two different screw configurations were tested for starch foam production. The first configuration consisted primarily of conveying elements, resulting in less shear. This setup did not efficiently mix the starch, additives, and water, leading to inconsistent foams with some solid blocks observed between foam strands, indicating inadequate mixing (Figure 3a). Despite its shortcomings, this configuration served as a baseline for adjusting extrusion parameters.

In the second screw configuration, additional kneading elements were incorporated to enhance mixing and increase torque (Figure 3c). The feed zone featured the largest single-pitch screw elements to rapidly convey starch and prevent material buildup. The screw pitch was then reduced to force materials downstream, and kneading elements were introduced at regular intervals to ensure efficient mixing of raw materials. Detailed information on both the screw configurations is provided in the supporting information. This improved configuration proved to be more effective in terms of mixing and torque and was used for all subsequent experiments. The foams produced with the second configuration exhibited greater consistency, with uniform structure and surface properties (Figure 3b). The details of both the screw configurations can be found in supplementary section (Table S1).

*Temperature profile:* Gelatinization is a process of breaking down the intermolecular bonds of starch molecules when exposed to water and heat. High amylose cornstarch gelatinizes at higher temperatures above 90–100 °C. Hence, the initial zones were maintained at lower temperatures, while the last four to five zones were set at temperatures higher than the gelatinization temperatures of starch. This strategy ensured that gelatinization occurred in the later zones just before the foaming stage. For other additives like PVOH and PVB, the temperatures were adjusted according to their melting temperatures. However, the temperature profile presented in Table 3 generally worked well for all formulations with minor adjustments. Sometimes, the initial two heat zone temperatures were temporarily increased to 80 °C to get the screws moving properly. This adjustment was necessary due to the presence of LDPE purge material in the extruder. A higher temperature was required to soften the LDPE present in the extruder and get the screws moving. Care was consistently taken to keep the initial zone temperatures below 90 °C to minimize the evaporation and loss of water from the feeding zone.

**Figure 4:**
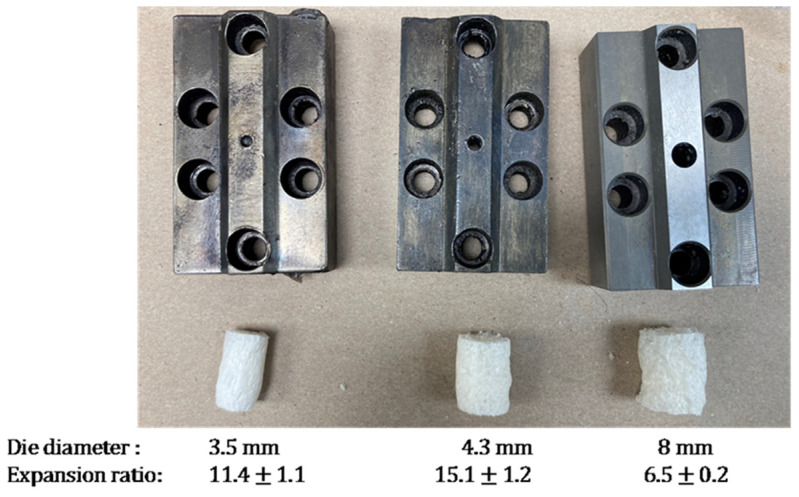
Three strand dies used for extrusion and their corresponding expansion ratios.

*Die diameter:* The density and expansion ratio of foams are influenced by the pressure developed at the extruder’s end. In addition to screw configuration and screw speed, the die type and diameter also significantly impact this pressure. A smaller die diameter results in higher pressure at the die face, resulting in a greater pressure drop and an increase in the expansion ratio. To investigate this effect, foams were produced using three-strand dies with diameters of 3.5 mm, 4.3 mm, and 8 mm, using formulations 6, 7, and 8 (Table 1), which included high amylose cornstarch, 10 wt.% PVOH, and 4 wt.% chitosan. Figure 4 shows the dies, the corresponding foams produced from them, and their expansion ratios. Expansion ratios for strand die foams were calculated as the ratio of the foams’ cross-sectional area to the cross-sectional area of the die. As expected, the smallest die yielded foams with the lowest density and highest expansion ratio. Conversely, as the die diameter increased, the density of the foams increased while the expansion ratio decreased. However, the smallest strand die (3.5 mm diameter) frequently encountered blockages at the die face, as the small opening became plugged with starch, leading to unstable runs. The 4.3 mm die produced more consistent and uniform foams, making it the preferred choice for subsequent runs. The largest strand die (8 mm diameter) resulted in foams with the highest density and lowest expansion ratio and was therefore not used further.

For all experiments, an optimized water content of 6–8%, a starch feeding rate of 150 g/min, and a screw speed of 180–200 rpm were employed. These values were based on previous research conducted by our group on starch foams, as detailed in the experimental section [4,13,25]. While these parameters were qualitatively adjusted to achieve optimal properties, detailed quantitative studies were not conducted.

### 3.2. Effect of Various Additives on Densities and Expansion Ratios of Foams

**Figure 5:**
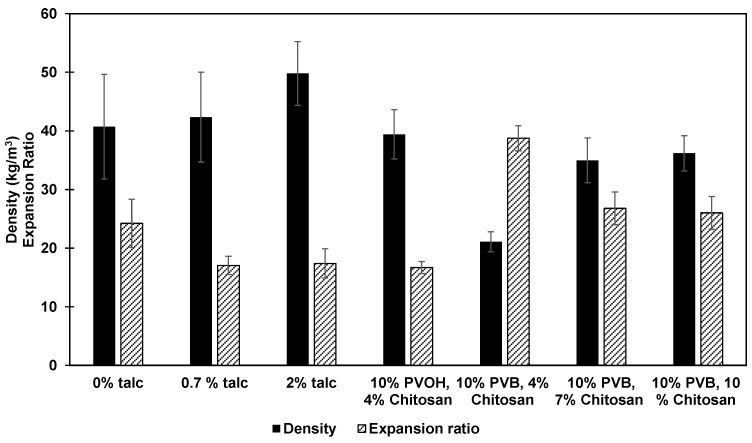
Effect of talc, PVOH, chitosan, and PVB on density and expansion ratio of starch foams.

Figure 5 illustrates the influence of talc, PVOH, PVB, and chitosan on the density and expansion ratios of starch foams. The data reveal that increasing talc content from 0% to 2% led to higher foam densities and reduced expansion ratios. Specifically, control foams made with only starch and water had a density of 40.7 ± 8.9 kg/m^3^ and an expansion ratio of 24.2 ± 4.2, which are slightly higher than values reported in other studies [11,13]. Control starch foams without any other additives were highly inconsistent in nature and very brittle, which could explain the higher standard deviation in the measurements. Increasing the talc content to 0.7% and 2% resulted in increased densities of 42.3 ± 7.3 kg/m^3^ and 49.7 ± 5.4 kg/m^3^, with corresponding expansion ratios of 17.1 ± 1.6 and 17.4 ± 2.5, respectively. The nucleating effect of talc on density and expansion ratios has been corroborated by numerous studies [9]. Besides increasing density and reducing the expansion ratio, talc addition also promoted the formation of a larger number of smaller cells, reducing their diameter. These results were further quantified and analyzed in the SEM studies detailed in the SEM section.

Incorporating PVOH and chitosan into the formulation resulted in a density comparable to the control foams (without any additives) 39.4 ± 4.7 kg/m^3^, but a lower expansion ratio of 16.7 ± 1.0. PVOH addition improved foam consistency, reduced brittleness, and smoothed the surface of the foam. This improvement can be attributed to the high molecular weight and water solubility of polyvinyl alcohol, which reduced water diffusivity, leading to more controlled expansion and, consequently, lower expansion ratios. The polymeric structure of PVOH and its proper interaction with starch molecules led to improved melt strength and, therefore, a decrease in the cell size of foams [28].

PVB, or polyvinyl butyraldehyde, is a resin commonly applied in automotive safety shields and adhesives for its ability to enhance flexibility and toughness. PVB is synthesized through the reaction of polyvinyl alcohol with butyraldehyde. Previous studies have shown that PVB improves the water barrier properties of paperboards and starch films [22,29]. Therefore, it was incorporated into starch foams to assess its impact on water solubility and moisture resistance. Our observations revealed that PVB also functioned as a plasticizer, significantly increasing the expansion ratio while decreasing the density of starch foams to 38.7 ± 2.1 and 21.1 ± 1.7 kg/m^3^, respectively. This effect is attributed to PVB reducing intermolecular hydrogen bonding between starch chains, which facilitated easier expansion and water penetration into the starch structure. Finally, the density of the foams increased from 21.1 to 36.2 kg/m^3^ as the chitosan content was raised from 4% to 10%, while the expansion ratio decreased from 38.75 to 26.05, as shown in Figure 5. This outcome suggests that chitosan exhibits a nucleating effect similar to that of talc, promoting smaller and more uniform cell formation. These effects were further investigated through detailed SEM analyses, as described in the corresponding section.

### 3.3. Cell Size and Cell Size Distributions

**Figure 6:**
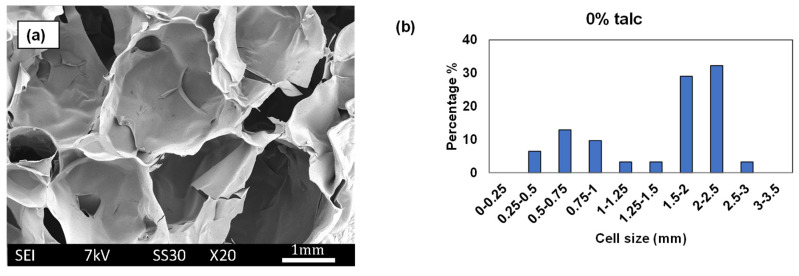
(**a**) SEM image of the fractured surface for control starch foam (no additives) and (**b**) cell size distribution of the foam.

**Figure 7:**
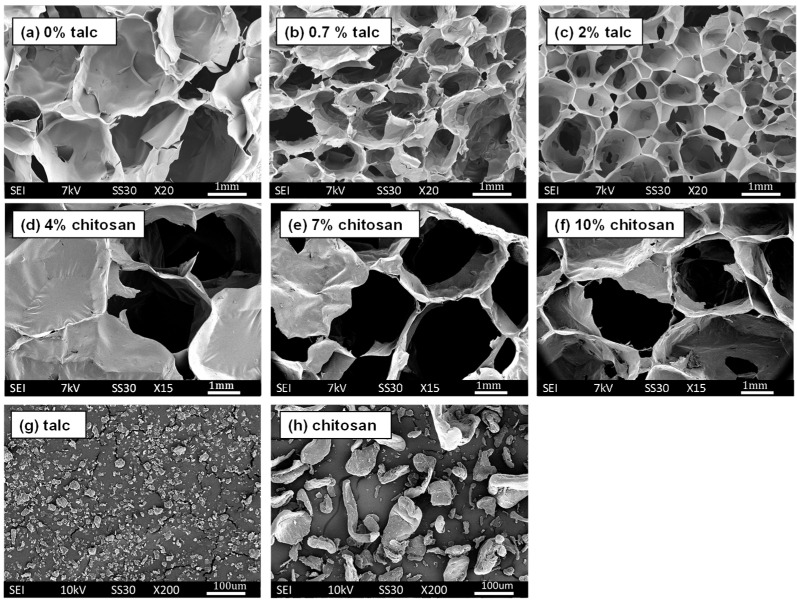
SEM images of starch foams containing (**a**) 0% talc, (**b**) 0.7% talc, (**c**) 2% talc, (**d**) 4% chitosan, 10% PVB, (**e**) 7% chitosan, 10% PVB, (**f**) 10% chitosan, 10% PVB, and SEM of, (**g**) talc, and (**h**) chitosan.

**Figure 8:**
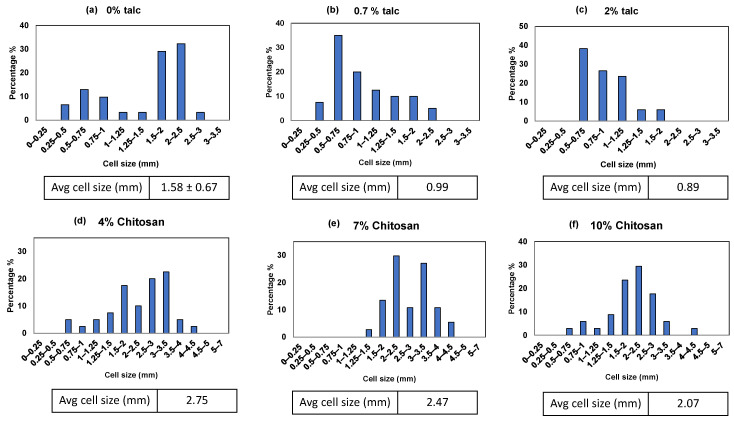
Cell size distribution of foams containing (**a**) 0% talc, (**b**) 0.7% talc, (**c**) 2% talc, (**d**) 4% chitosan, 10% PVB, (**e**) 7% chitosan, 10% PVB, and (**f**) 10% chitosan, 10% PVB.

SEM images (Figure 6) and ImageJ analysis of starch foams produced with only water (no additives) revealed a highly irregular cell structure with a broad cell size distribution. The average cell size for this formulation was measured at 1.58 mm with a standard deviation of 0.67 mm. This variability is attributed to the absence of nucleating agents or stabilizers, resulting in uneven foam expansion during extrusion. In contrast, foams formulated with increasing talc content demonstrated an improvement in cell uniformity. As illustrated in Figure 7b and Figure 7c, corresponding to 0.7% and 2% talc, higher talc concentrations yielded a greater number of smaller, well-distributed cells. The average cell size decreased to 0.99 mm and 0.89 mm, respectively. The narrowing of cell size distribution, confirmed by the normalized graphs in Figure 8a–c, highlights talc’s effectiveness as a nucleating agent. By facilitating uniform bubble formation during foaming, talc reduces cell size and improves the foam’s overall consistency.

Incorporating chitosan also demonstrated a nucleating effect, although less pronounced than talc (see Figure 7d compared to Figure 7e,f). This effect is quantified in the normalized graphs where the percentage of cells within each size range was plotted for all formulations. As shown in Figure 7d–f, increasing chitosan content led to a noticeable shift in the cell size distribution toward smaller sizes. The average cell size for the 4% chitosan formulation was 2.75 mm, decreasing to 2.47 mm and 2.07 mm for 7% and 10% chitosan, respectively. It is worth noting that these foams contained PVB as well. PVB acted as a plasticizer and increased the cell size and expansion ratios of the foams considerably as compared to control foams and foams with talc. Higher amounts of chitosan were required for achieving similar nucleating effect as talc. This difference in the impact of talc vs. chitosan can be attributed to the larger and more non-uniform chitosan particles compared to talc, as seen in their SEM images (Figure 7g,h). According to McClurg’s qualitative guidelines [30], ideal nucleating agents should possess uniform size, geometry, and surface properties and should be easily dispersible. Leung et al. [31] further suggested that the geometry of the nucleating agent influences its efficiency. Talc, with its smaller and scaly particles, likely explains its superior nucleating efficiency compared to the larger, irregularly sized chitosan particles. González-Núñez et al. studied chitosan as a nucleating agent for thermoplastic foams, and they found that, for every nucleating agent, there is a “critical concentration” at which the cell size and distribution are most uniform. And the critical concentration depends on the size of the particles. The larger the particle size, the higher the percentage required for the nucleating effect [32]. Thus, the lack of impact observed with chitosan in this study could be due to insufficient concentrations, which were not tested due to cost constraints.

### 3.4. Evaluating Water Resistance in Starch Foams

**Figure 9:**
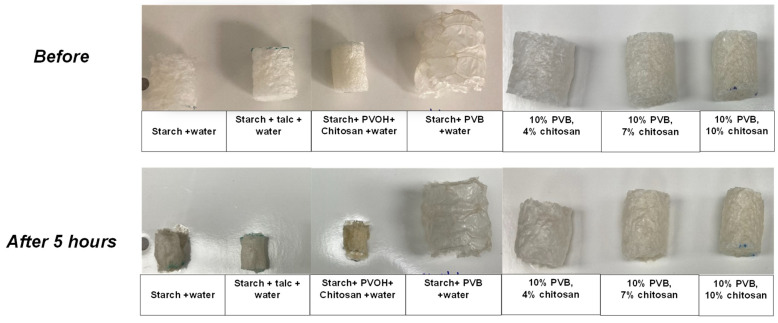
Effect of various additives on water penetration of foams.

To evaluate water resistance, starch foam noodles were immersed in water, removed, and left undisturbed for 5 h. Photographs were taken before and after immersion to evaluate the impact of water exposure on the foam samples, as shown in Figure 9. The results indicated that control foams, as well as those containing talc and PVOH, experienced significant disintegration and shrinkage. In contrast, foams with chitosan or PVB demonstrated improved water resistance, with minimal disintegration and shrinkage.

**Figure 10:**
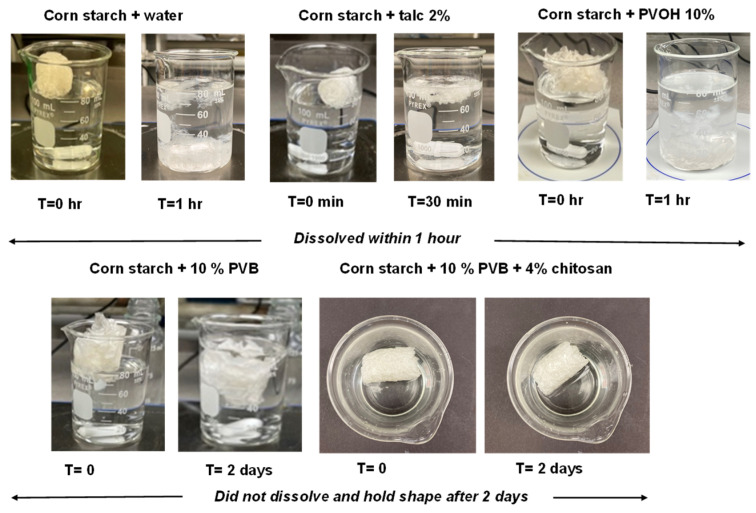
Water solubility testing for starch foams.

Further analysis involved testing the water solubility of these foams. Control foams and those containing PVOH dissolved rapidly within minutes of water exposure, as shown in Figure 10. In contrast, foams containing PVB exhibited a marked reduction in solubility. Specifically, foams with only PVB or a combination of chitosan and PVB did not dissolve in water, as illustrated in Figure 10. This enhanced water resistance can be attributed to the hydrophobic nature of PVB and its film-forming and binding properties, which contribute to the cohesion of the foam structure, preventing dissolution for up to two days.

### 3.5. Analysis of Moisture Sensitivity Using Peleg Model

**Figure 11:**
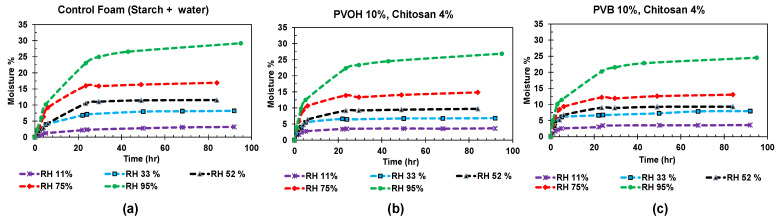
Moisture absorption curves for starch foams in different RH conditions (**a**) control foams (starch + water), (**b**) PVOH + chitosan + water, and (**c**) starch + PVB + chitosan.

The moisture absorption behavior of starch foams with various additives is illustrated in Figure 11, which presents results at room temperature under different relative humidity (RH) conditions.

**Figure 12:**
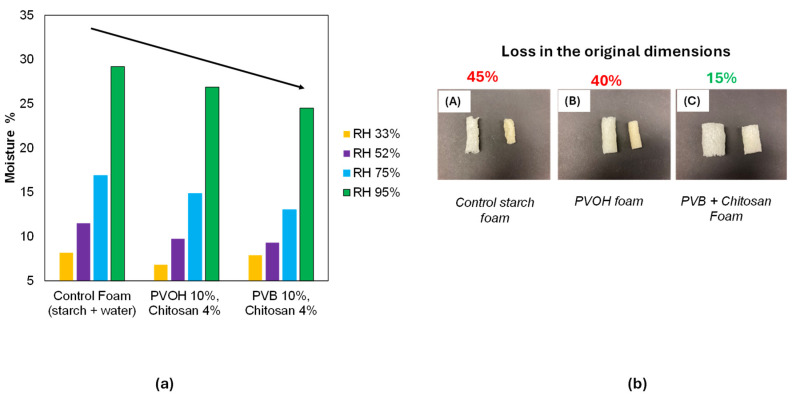
(**a**) moisture content at equilibrium for different foam formulations at 95% RH. (**b**) shrinkage observed in foams when placed in 95% RH environment.

All samples demonstrated typical adsorption behavior, characterized by an initially rapid moisture uptake that gradually slowed down, reaching equilibrium after approximately 40 h. Figure 12a provides an alternative representation of these findings. The equilibrium moisture content of the foams under RH conditions of 33%, 52%, 75%, and 95% was plotted for three different foam formulations. It was evident that the equilibrium moisture levels decreased for all formulations across the RH environments. The most pronounced reduction was observed in the 95% RH condition, where the equilibrium moisture content after 80 h decreased from 29% (by weight) for the control foams to 24% for foams containing PVB and chitosan.

Dimensional stability, defined as minimal dimensional loss, is a critical factor for packaging applications. Regarding physical stability, these foams became brittle in low RH environments (11% and 33%). In the high RH environment (95%), noticeable shrinkage occurred in the control foams and those with PVOH, which lost 40–45% of their original dimensions (Figure 12b, first and second images). In contrast, foams containing PVB and chitosan exhibited significantly better dimensional stability, with only a 15% reduction in dimensions (Figure 12b, third image).

From the shape of the moisture absorption curves, it was apparent that the moisture absorption was directly proportional to the RH. The moisture sorption kinetics was further modeled and quantified using the Peleg equation [33,34], given as:

$$M\left(t\right)=M_{0}+\frac{t}{k_{1}+k_{2}t}$$

where *M*(*t*) is the moisture content at any time *t* and *M*_0_ is the initial moisture content of the sample. Peleg model’s advantage lies in its ability to predict moisture sorption kinetics using short-term experimental data. Here, *k*_1_ represents the mass transfer rate constant (with lower values indicating higher initial absorption rates), and *k*_2_ represents the maximum absorption capacity constant (with lower values indicating higher maximum absorption). The Peleg model parameters and goodness of fit (R^2^) were calculated using Minitab, as summarized in Table 4.

The data in Table 4 indicates that the *k*_1_ and *k*_2_ values were lowest for the control starch foams, indicating their rapid water absorption rate and high water-absorbing capacity. The addition of PVOH and chitosan resulted in slightly higher *k*_1_ and *k*_2_ values, likely due to the hydrophobic nature of chitosan [35,36]. The addition of PVB to minimize the water sensitivity was found to be extremely effective. Foams modified with PVB exhibited the highest *k*_1_ and *k*_2_ values in almost all cases, corresponding to the lowest rate of water absorption and the lowest moisture absorption capacity among the samples.

The variations in *k*_1_ and *k*_2_ values are directly related to the physical stability of the foams. Control starch foams, with lower *k*_1_ and *k*_2_ values, exhibited poor stability due to their rapid and excessive water uptake, which can lead to swelling, dimensional instability, surface disintegration, and rapid dissolution in water, as observed in Section 3.4. In contrast, PVB-modified foams, with higher *k*_1_ and *k*_2_ values, demonstrated enhanced resistance to moisture absorption, correlating with their superior dimensional stability under high RH conditions. This improved dimensional stability, combined with reduced moisture absorption, highlights the potential of PVB and chitosan-enhanced foams for packaging applications in high-humidity environments.

### 3.6. Effect of Chitosan and PVB on Surface Properties of Starch Foams

**Figure 13:**
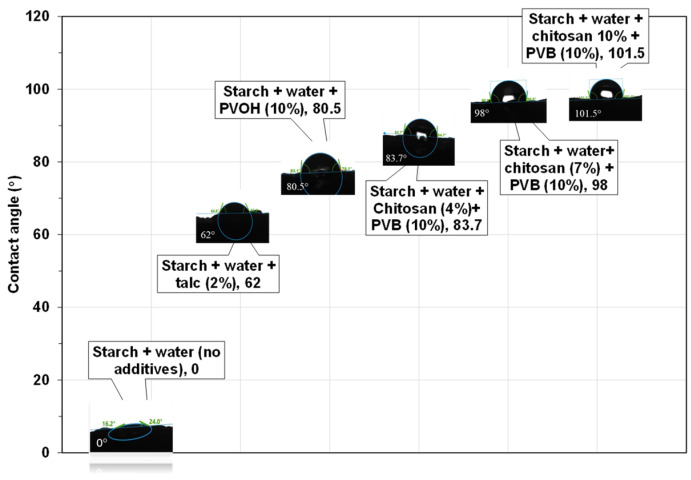
Effect of different additives on surface hydrophobicity of foams.

*Contact angle measurements:* Starch foam’s surface hydrophobicity water was studied with contact angle measurements. Figure 13 shows the contact angles of water droplets on different starch foam surfaces with various additives after 30 s.

For the control cornstarch foams containing only starch and water, it was observed that the water droplets did not stay on the surface. They were rapidly absorbed into the foam due to the hydrophilic starch surface. The addition of talc increased the hydrophobicity of the foam surface, likely due to the inherently hydrophobic properties of talc [37]. However, previous water solubility and moisture absorption tests demonstrated that talc alone was insufficient to impart significant moisture resistance to the foams. Incorporating PVOH into the foams resulted in a smoother surface compared to the control foams. Water droplets remained on the PVOH-containing foams longer, with an initial contact angle of 80.5°. However, the contact angle decreased rapidly with time, and the droplets were absorbed within approximately 3 min.

The addition of chitosan and PVB resulted in a slightly higher contact angle (83.7°). In these formulations, water droplets remained on the foam surface for an extended period without being absorbed, indicating enhanced surface hydrophobicity. This synergistic effect of chitosan and PVB is attributed to the inherent hydrophobicity of PVB and the interaction between the amino groups of chitosan and the hydroxyl groups of starch, which likely stabilized the hydrophilic starch matrix and reduced the number of polar groups available for bonding with water [38]. With increasing chitosan content from 4% to 10%, a steady increase in contact angle up to 101.5° was observed. This increase is likely due to the hydrogen bonding between the polar groups of chitosan and starch, reducing the availability of polar groups on the foam surface for interaction with water.

*Confocal microscopy:* The migration of chitosan to the surface of the foams was examined using CLSM. Previous studies have utilized various fluorescent labels, such as FITC [39] and rhodamine B isothiocyanate (RBITC) [40] for characterizing chitosan with CLSM. In this study, FITC was employed for green fluorescent staining of chitosan.

**Figure 14:**
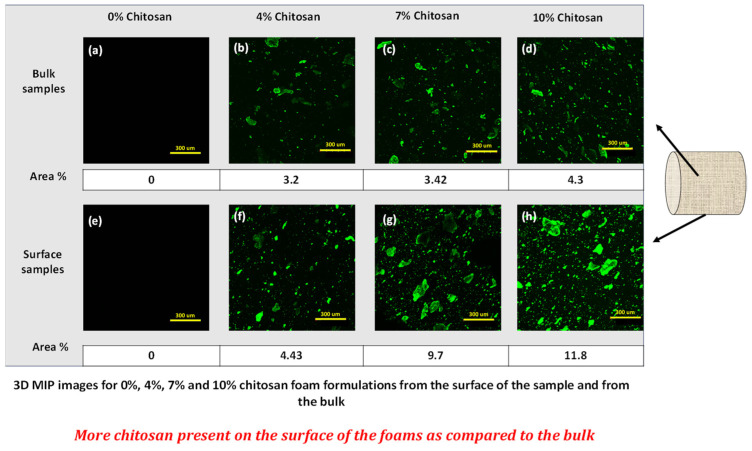
CLSM 3D MIP images for 0%, 4%, 7%, and 10% chitosan foam formulations from the bulk of the sample (**a**–**d**) and from the surface (**e**–**h**). Schematic for interaction between the amino or OH groups of chitosan and OH groups of starch.

The migration of chitosan to the foam surface is believed to contribute to the increased hydrophobicity observed in these foams. To investigate this phenomenon, the distribution of chitosan in the bulk (middle part) and on the surface of the foams was analyzed using CLSM. The foams were cut in thin slices from both the inner bulk (central portion) and the surface of the foam. Then they were stained and mounted on the slides, as described in the experimental section. The relative amounts of chitosan in the bulk and on the surface of the foams were compared and quantified using ImageJ analysis. Figure 14 presents the 3D maximum intensity projection (MIP) images for foam formulations with 0%, 4%, 7%, and 10% chitosan from both the surface and bulk of the samples. In all cases, a higher concentration of chitosan was clearly observed on the foam surface compared to the bulk. Quantitative analysis of the green fluorescent-stained chitosan further supported these observations. For instance, in samples containing 7% chitosan, the area of green fluorescence in the bulk was measured at 3.42%, whereas on the surface, it was significantly higher at 9.7%. Similar trends were observed for foams with 4% and 10% chitosan content. The findings corroborate those of Dang et al. (2016), where they reported that chitosan migrates to the surface of films, forming hydrogen bonds with starch molecules and increasing the surface hydrophobicity [24]. A similar migration pattern was confirmed in the foams studied here. This migration likely occurred during the cooling process post-extrusion or during storage. This also explains the increased hydrophobicity of the foam surface as observed from contact angle measurements.

### 3.7. Compressive Strength and Resiliency

**Figure 15:**
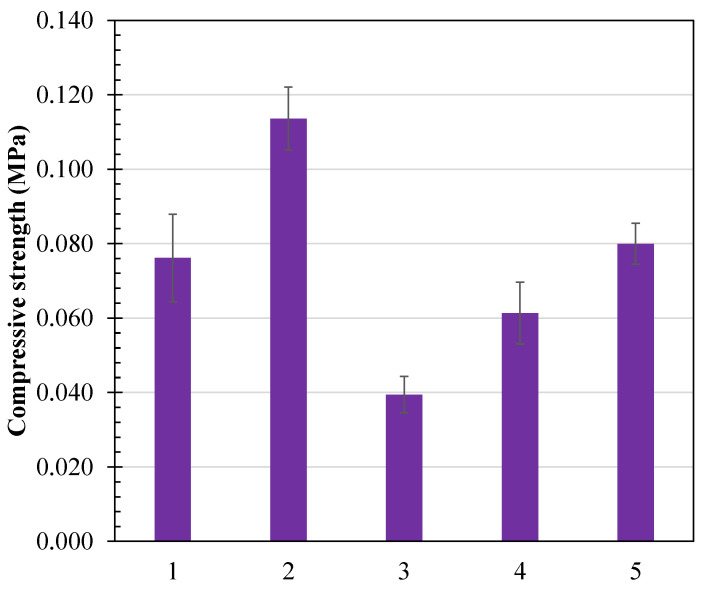
Compressive strength of starch foams (1) Control foams (starch + water), (2) control foams (PVOH), (3) 10% PVB, 4% chitosan, (4) 10% PVB, 7% chitosan, and (5) 10% PVB, 10% chitosan.

Compressive strength is the measure of the ability of foam to deform under load. Figure 15 shows the effect of various additives on the compressive strength, with detailed results listed in Table 5. Denser foams tend to have thicker walls and hence resist deformation better and thus have a higher compressive strength. This trend is clearly observed in the control foam sample (40.7 kg/m^3^, 0.076 MPa) compared to the foams containing PVB, which exhibit lower density and compressive strength (e.g., 21.1 kg/m^3^, 0.040 MPa for 10% PVB, and 4% chitosan). Since the addition of PVB reduced the density of foams considerably, as explained previously, the compressive strength was also found to be less for these foams. Interestingly, as the chitosan content increased from 4% to 7% to 10% in PVB-modified foams, the density also increased progressively (e.g., 21.1–35.7 kg/m^3^), leading to improved compressive strength (e.g., 0.040–0.080 MPa). This suggests that by adjusting the levels of PVB and chitosan, the density and mechanical properties of the foam can be effectively tuned to meet specific requirements.

Control foams displayed a resilience of 64.5%, indicating a balance of elasticity and firmness. PVB-modified foams exhibited resilience values ranging from 60.9% to 64.8%, depending on chitosan content, suggesting that PVB enhances flexibility while maintaining a reasonable degree of recovery. However, PVOH-modified foams had slightly lower resilience (54.8%), likely due to their denser structure, which restricts rapid recovery. These results underlined the importance of tunability for tailoring starch foam properties according to the required applications.

### 3.8. Biodegradability of the Foams in Aqueous Environment

**Figure 16:**
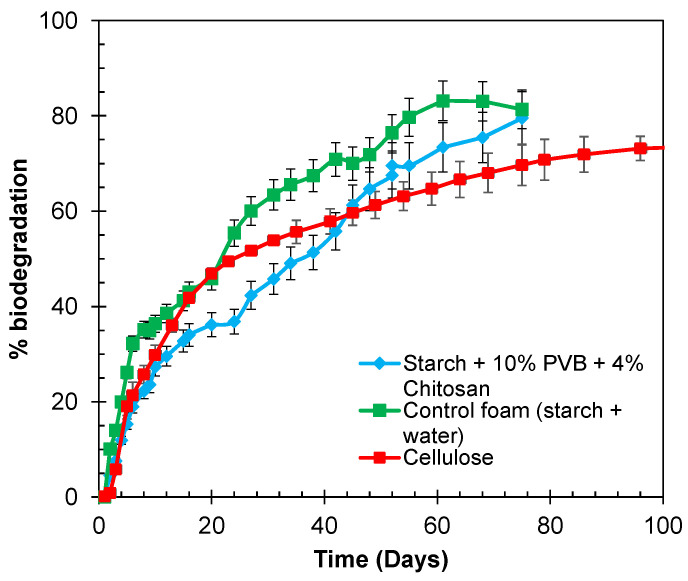
Aqueous biodegradation curves for control starch foam, PVB, and chitosan containing foam and cellulose.

The impact of adding PVB and chitosan on the end-of-life biodegradation of foams was assessed using an aqueous biodegradation test, following ISO 14852 at 30 °C. Two foam samples were analyzed: a control foam containing only starch and a foam with 4% chitosan and 10% PVB. Cellulose served as the positive control. The percentage of biodegradation was calculated by subtracting the CO_2_ evolved from the samples from the cumulative CO_2_ evolved the blanks, as described in the experimental section. Figure 16 illustrates the percent biodegradation over time for the three samples. The biodegradation rates of the three samples were found to be very similar. Despite the presence of PVB and chitosan, the foams achieved approximately 80% biodegradation within 80 days, which is a favorable outcome. Initially, the foams containing PVB and chitosan showed a slower biodegradation rate. This delay is likely attributed to chitosan’s antimicrobial properties and the hydrogen bonding between starch and chitosan, which may have reduced the starch matrix’s hydrophilicity and slowed the biodegradation process [41]. However, after the initial incubation period, the rate of biodegradation accelerated, reaching the same 80% biodegradation level as the control foam. While PVB itself is not inherently biodegradable, its presence did not appear to significantly hinder the biodegradation of the rest of the foam formulation, including the starch matrix. This suggests that the non-biodegradable PVB remained largely inert during the biodegradation process, allowing the biodegradable components to degrade efficiently. However, it is important to note that due to the inclusion of PVB, the foams may not achieve complete (100%) biodegradation. Nonetheless, the modifications using PVB and chitosan enhanced the moisture resistance of the foams without compromising the overall biodegradability of the starch-based components, maintaining their environmental compatibility for sustainable applications.

## 4. Conclusions

This study successfully demonstrates the development of moisture-resistant, partially biodegradable starch foams using an optimized one-step extrusion-foaming process, enhanced with PVB and chitosan. The extrusion parameters—including temperature, screw configuration, die diameter, water content, and feeding rates—were optimized to achieve foams with the lowest density and controlled expansion. PVB acted as a plasticizer, producing foams with the lowest density (21 kg/m^3^) and highest expansion ratio (38.7), while chitosan functioned as a nucleating agent, reducing the cell size and promoting a more uniform cell size distribution. The incorporation of various percentages of chitosan allowed for fine-tuning the densities and cell size distribution of the foams. The combined addition of PVB and chitosan significantly improved the moisture resistance of the starch foams, resulting in superior performance in water insolubility, dimensional stability, and surface hydrophobicity. CLSM imaging confirmed the migration of chitosan to the surface of the foams, making the surface hydrophobic. These enhanced starch foams hold significant potential for various applications, including packaging and insulation, where long-term humidity resistance is required. Their use could substantially reduce the carbon footprint associated with conventional polyethylene or polystyrene foams, offering an environmentally responsible end-of-life solution. This research underscores the feasibility of creating sustainable materials that balance performance with biodegradability, addressing both functional requirements and environmental concerns.

## Figures and Tables

**Table 1:** Extrusion runs with various additives.

Formulation #	Starch Type	talc (%)	Chitosan (%)	PVOH (%)	PVB (%)	Die Diameter (mm)
***Variation in talc content***						
**1**	High amylose cornstarch	0	-	-	-	4.3
**2**	High amylose cornstarch	0.7	-	-	-	4.3
**3**	High amylose cornstarch	2	-	-	-	4.3
***Using PVOH and chitosan (variation in die diameter)***						
**4**	High amylose cornstarch	-	-	10	-	4.3
**5**	High amylose cornstarch	-	4	10	-	4.3
**6**	High amylose cornstarch	-	4	10	-	3.5
**7**	High amylose cornstarch	-	4	10	-	8
***Using PVB and Variation in chitosan content***						
**8**	High amylose cornstarch	-	0	-	10	4.3
**9**	High amylose cornstarch	-	4	-	10	4.3
**10**	High amylose cornstarch	-	7	-	10	4.3
**11**	High amylose cornstarch	-	10	-	10	4.3

**Table 2:** Different salt solutions for relative humidity.

Salt	RH
**Lithium chloride**	11%
**Magnesium chloride**	33%
**Magnesium nitrate**	52%
**Sodium chloride**	75%
**Potassium Nitrate**	95%

**Table 3:** General temperature profile for starch foam extrusion.

Zone	1	2	3	4	5	6	7	8	9	Die
**Temperature (°C)**	40	60	80	90	100	110	120	130	140	140

**Table 4:** Parameters of Peleg model for kinetics of moisture absorption of starch foams.

Sample	RH	*k*_1_	*k*_2_	R^2^
**Control foam**	LiCl = 11%	0.444	0.249	0.984
**PvOH 10%, chitosan 4%**		0.460	0.270	0.988
**PVB 10%, chitosan 4%**		0.615	0.279	0.987
**Control foam**	MgCl_2_ = 33%	0.114	0.131	0.998
**PVOH 10%, chitosan 4%**		0.302	0.141	0.991
**PVB 10%, chitosan 4%**		0.147	0.136	0.994
**Control foam**	MgNO_3_ = 52%	0.100	0.093	0.997
**PVOH 10%, chitosan 4%**		0.410	0.095	0.996
**PVB 10%, chitosan 4%**		0.352	0.100	0.994
**Control foam**	NaCl = 75%	0.129	0.050	0.994
**PVOH 10%, chitosan 4%**		0.181	0.066	0.884
**PVB 10%, chitosan 4%**		0.185	0.075	0.998
**Control foam**	KNO_3_ = 95%	0.070	0.037	0.988
**PVOH 10%, chitosan 4%**		0.215	0.035	0.999
**PVB 10%, chitosan 4%**		0.206	0.039	0.995

**Table 5:** Physio-mechanical properties of extruded starch foams.

Sample	Density (kg/m^3^)	Expansion Ratio	Compressive Strength (MPa)	Resiliency (%)
Control foam	40.7 ± 8.9	46.6 ± 7.9	0.076 ± 0.01	64.5 ± 3.7
PVOH 10%, chitosan 4%	45.7 ± 2.3	27.5 ± 0.9	0.114 ± 0.01	54.8 ± 1.5
PVB 10%, chitosan 4%	21.1 ± 1.7	74.5 ± 3.9	0.040 ± 0.005	64.8 ± 2.3
PVB 10%, chitosan 7%	35.0 ± 3.8	51.1 ± 5.5	0.0614 ± 0.008	62.4 ± 1.8
PVB 10%, chitosan 10%	35.7 ± 3.0	50.1 ± 5.7	0.0800 ± 0.005	60.9 ± 2.8

## Data Availability

The data presented in this study are available on request from the corresponding author.

## Supplementary Materials

The following supporting information can be downloaded at: https://www.mdpi.com/article/10.3390/polym16233402/s1, Table S1: Screw configurations used for starch foam extrusion.
