# Supplementary material for: Humidity Resistant Biodegradable Starch Foams Reinforced with Polyvinyl Butyral (PVB) and Chitosan
Source: Polymers (Basel). 2024 Dec 3;16(23):3402. doi: 10.3390/polym16233402 (PMC11644289; doi:10.3390/polym16233402)
Supplement: Supplementary file 1 [file polymers-16-03402-s001.zip › polymers-3311316-supplementary.pdf]

Supporting information

Humidity-Resistant Biodegradable Starch Foams Reinforced with Polyvinyl Butyral (PVB) and Chitosan

Apoorva Kulkarni[1], Jacob Emrich[1], Ramani Narayan[1]*

[1] Department of Chemical Engineering and Materials Science, Michigan State University, East Lansing, MI 48824, USA

** Author to whom correspondence should be addressed*

Email : narayan@msu.edu Phone: +1-517-303-7316

Table S1: Screw configurations used for starch foam extrusion.

#	Screw config #1	Screw config #2
1	28/14	28/14
2	60/60	60/60
3	60/60	60/60
4	60/60	60/60
5	42/42	42/42
6	42/42	28/28
7	28/28	28/28
8	20/20	20/20
9	20/20	20/20
10	20/20	KB 45/5/14
11	20/20	KB 45/5/14
12	KB 45/5/28	KB 45/5/14

Supporting information

13	KB 90/5/28	KB 45/5/20
14	KB 90/5/28	KB 45/5/20
15	KB 45/5/14	60/60
16	KB 45/5/14	42/42
17	28/28	28/28
18	28/28	28/28
19	28/28	20/20
20	20/20	KB 45/5/42
21	20/20	KB 45/5/42
22	20/20	KB 45/5/42
23	20/20	KB 45/5/42
24	20/20	60/60
25	20/20	42/42
26	20/20	28/28
27	KB 45/5/14	28/28
28	KB 45/5/14	20/20
29	KB 90/5/28	KB 45/5/14
30	KB 90/5/28	KB 45/5/14
31	28/28	KB 90/5/28
32	28/28	KB 90/5/28
33	20/20	60/60
34	42/42	42/42
35	42/42	42/42
36	42/42	28/28
37	42/42	28/28
38	42/42	28/14

Supporting information

39	28/28	20/20
40	28/28	KB 90/5/28
41	20/20	20/20
42		20/20
43		20/20
